# Comparative transcriptomic analysis of the gene expression and underlying molecular mechanism of submergence stress response in orchardgrass roots

**DOI:** 10.3389/fpls.2022.1104755

**Published:** 2023-01-10

**Authors:** Minghao Qu, Yuqian Zheng, Lei Bi, Xingyun Yang, Panpan Shang, Xiaoli Zhou, Bing Zeng, Bingna Shen, Wenwen Li, Yan Fan, Bing Zeng

**Affiliations:** ^1^ College of Animal Science and Technology, Southwest University, Chongqing, China; ^2^ Institute of Prataculture, Chongqing Academy of Animal Science, Chongqing, China; ^3^ Chongqing University Herbivore Engineering Research Center, Chongqing, China

**Keywords:** *Dactylis glomerata* L., transcriptome, submergence stress, differentially expressed genes, molecular mechanisms

## Abstract

**Introduction:**

Submergence stress creates a hypoxic environment. Roots are the first plant organ to face these low-oxygen conditions, which causes damage and affects the plant growth and yield. Orchardgrass (*Dactylis glomerata* L.) is one of the most important cold-season forage grasses globally. However, their submergence stress-induced gene expression and the underlying molecular mechanisms of orchardgrass roots are still unknown.

**Methods:**

Using the submergence-tolerant ‘Dianbei’ and submergence-sensitive ‘Anba’, the transcriptomic analysis of orchardgrass roots at different time points of submergence stress (0 h, 8 h, and 24 h) was performed.

**Results:**

We obtained 118.82Gb clean data by RNA-Seq. As compared with the control, a total of 6663 and 9857 differentially expressed genes (DEGs) were detected in Dianbei, while 7894 and 11215 DEGs were detected in Anba at 8 h and 24 h post-submergence-stress, respectively. Gene Ontology (GO) enrichment analysis obtained 986 terms, while Kyoto Encyclopedia of Genes and Genomes (KEGG) enrichment analysis obtained 123 pathways. Among them, the DEGs in plant hormones, mitogen-activated protein kinase (MAPK) and Ca^2+^ signal transduction were significantly differentially expressed in Dianbei, but not in Anba.

**Discussion:**

This study was the first to molecularly elucidate the submergence stress tolerance in the roots of two orchardgrass cultivars. These findings not only enhanced our understanding of the orchardgrass submergence tolerance, but also provided a theoretical basis 36 for the cultivation of submergence-tolerant forage varieties.

## Introduction

Water is essential for plants, but excessive water, waterlogging or flooding can cause stress by preventing the exchange of gases between the soil and the atmosphere, which negatively impacts plant growth and development (Striker and Colmer, 2016; Wang et al., 2017). Although oxygen accounts for ~20% in the air, it has extremely low solubility in water. The diffusion rate of oxygen in flooded soil is 10^4^ times slower than in air. Furthermore, respiration of soil microorganisms and plant roots rapidly depletes the oxygen level (Kaur et al., 2021). Root is the first organ facing the decline of oxygen tension. Since they can’t produce oxygen through photosynthesis, it will soon face hypoxia under submergence stress, ultimately causing more damage to the roots (Panozzo et al., 2019). Plants with submergence-tolerant depend heavily on multiple morphological and physiological changes that are regulated by different genes. RNA-seq has been widely used in plant field in recent years, especially in the research of plant resistance (Li et al., 2018). Transcriptome analysis revealed that ethylene and ABA synthesis in *Phalaris arundinacea* were inhibited by waterlogging stress (Wang et al., 2021). In deep-water rice (*Oryza sativa*) cultivar, the elevated ethylene levels promoted the expression of *SK1/SK2*, and reduced abscisic acid (ABA) biosynthesis, which further increased gibberellin (GA) content and induced cell elongation (Zhou et al., 2020). In maize (*Zea mays*), invertase and hexokinase expression were up-regulated under waterlogged stress in roots, providing more energy for self-stabilization (Arora et al., 2017). The insufficient energy resulting from the stress-induced oxygen shortage triggers reactive oxygen species (ROS) formation (Yeung et al., 2018; Anee et al., 2019). The hypoxic environment inhibits ATP production, restricts mitochondrial respiration, and alters the ratios of ATP to ADP and ATP to AMP (Sairam et al., 2008). Submergence causes plants to grow under hypoxic conditions, which limits the gas exchange capability of plants and impairs the metabolic balance and nutrient transport of plant roots, resulting in an ‘energy crisis’ (Herzog et al., 2016; Zhou et al., 2020). In a word, submergence can cause plant morphological damage, increasing the susceptibility of plant to diseases and insect pests, and even cause plant death (Phukan et al., 2016). RNA-seq can be used to study the molecular mechanism of plant response to submergence stress.

Grasslands are distributed worldwide except for Antarctica. The grassland covers an area of 52,544,000 km^2^, which is 40.5% of the world’s land area. It is the second largest land type for human habitation after agricultural land (Karunarathna et al., 2021). Orchardgrass is a cold-season, and perennial forage grass native to Eurasia and North Africa. As one of the four major forages in the world, it can be used as pasture or hay grass and has gained good ecological and economic benefits in China (Hirata et al., 2011). As an important forage grass for cultivating livestock worldwide, orchardgrass has the advantages of fast growth, high yield, good palatability, rich nutrition and strong stress resistance (Feng et al., 2018). Orchardgrass is mainly distributed in Sichuan, Chongqing, Yunnan, Guizhou, and other regions in China. Because of rich germplasm resources, orchardgrass has been widely planted and applied. It is an important species for land management and restoration in Southwest China (Peng et al., 2008). Therefore, in the world of increasing forage resources demand, submergence-tolerant is a key for orchardgrass breeding programs.

In recent years, with the rapid development of the next-generation sequencing (NGS) technology and the assembly of orchardgrass genome, a better platform for evaluating molecular and genetic information of orchardgrass is available (McCombie et al., 2019; Huang et al., 2020). *WRKY*, *GRAs*, and C2H2-type zinc finger gene family have previously been studied in orchardgrass (Xu et al., 2020; Ren et al., 2021; Shuai et al., 2022). Studies on biotic and abiotic stress reports of orchardgrass and other cold-season forage grasses had mainly focused on drought, high temperature, and rust (Huang et al., 2015; Ji et al., 2018; Yan et al., 2013, 2016; Hu et al., 2014; Sun et al., 2020). Although there are some studies on submergence stress in orchardgrass (Klaas et al., 2019; Qiao et al., 2020; Zeng et al., 2020), but it’s underlying submergence stress mechanisms in roots have not been reported. In the current study, the aim of this study was to understand the gene expression and underlying molecular mechanism of different submergence stress times response in two orchardgrass cultivars roots by RNA-seq. These findings can provide a basis for further study of the submergence tolerance mechanism of orchardgrass.

## Materials and methods

### Plant materials and growth conditions

In this experiment, the submergence-tolerant ‘Dianbei’ (DB) and submergence-sensitive ‘Anba’ (AB) were selected as test materials. The seeds of orchardgrass were obtained from the College of Animal Science and Technology of Southwest University, China. The seeds of orchardgrass were germinated in culture dish. Orchardgrass with the same growth trend were transplanted to the flowerpot (diameter 15.0 cm, height 13.5 cm) containing vermiculite, vegetative soil, and perlite (1:3:1, v/v/v). The orchardgrass cultivars were grown in an incubator with humidity of 70-85%, temperature of 22/15°C (day/night), photoperiod 14 h/10 h (day/night), and 5000 lux light intensity (Xu et al., 2017). Subsequently, the plants were irrigated with 1/2 Hoagland nutrient solution twice a week.

At the 3-4 leaf stage, the submergence treatment was started. Well-grown orchardgrass were selected for testing. Orchardgrass were placed in a water tank (length 80 cm, width 57 cm, height 50 cm), and water was added until the orchardgrass were completely submerged. Root phenotype and root length were used as growth characteristics data. The root length was measured and photographed at 0 h, 8 h, 24 h, 48 h and 72 h after submergence stress, and each treatment was repeated three times. Samples were taken at 0 h, 8 h, and 24 h under submergence stress, frozen in liquid nitrogen immediately, and stored at −80°C for further analysis. Each treatment was repeated for three times, with a total of 18 samples.

### RNA extraction and sequencing

Total RNA was extracted from roots at each time point (three biological replicates per treatment) using Trizol reagent (Invitrogen) according to the manufacture’s protocol. Total amounts and integrity of RNA were assessed using the RNA Nano 6000 Assay Kit of the Bioanalyzer 2100 system (Agilent Technologies, CA, USA). Samples with a RIN > 6.5 were used in RNA-seq library preparation. Total RNA was used as the input material for the mRNA sample preparations. The mRNA was purified from the total RNA by using poly-T oligo-attached magnetic beads. Fragmentation was carried out using divalent cations under elevated temperature in First Strand Synthesis Reaction Buffer(5X). First strand cDNA was synthesized using random hexamer primer and M-MuLV Reverse Transcriptase, then use RNaseH to degrade the RNA.Second strand cDNA synthesis was subsequently performed using DNA Polymerase I and dNTP. Remaining overhangs were converted into blunt ends *via* exonuclease/polymerase activities. After adenylation of 3’ ends of DNA fragments, Adaptor with hairpin loop structure were ligated to prepare for hybridization. In order to select cDNA fragments of preferentially 370~420 bp in length, the library fragments were purified with AMPure XP system (Beckman Coulter, Beverly, USA). Then PCR amplification, the PCR product was purified by AMPure XP beads, and the library was finally obtained. After the construction of the library, the library was initially quantified by Qubit2.0. After the insert size met the expectation, qRT-PCR was used to accurately quantify the effective concentration of the library (the effective concentration of the library was higher than that of 2nM) to ensure the quality of the library.

After the library was qualified, the different libraries were pooling according to the effective concentration and the target amount of data of the machine, then being sequenced by the Illumina NovaSeq 6000 (Illumina, San Diego, CA, USA). The end reading of 150 bp pairing was generated. Library construction and transcriptome sequencing were conducted by the Novogene Bioinformatics Institute (Beijing, China). All reads have been deposited in the sequence read archive (SRA) under the accession numbers PRJNA897027 and PRJNA896863.

### RNA-Seq data analysis

The raw data size was at least 6 Gb for each sample. The end reading of 150 bp pairing was generated. Clean reads were obtained by removing reads containing adapter, reads containing N base, and low quality reads from raw reads. Reference genome and gene model annotation files were downloaded from the genome website (https://orchardgrassgenome.sicau.edu.cn/) (Huang et al., 2020). Building of the reference genome index and alignment of the paired-end clean reads were done using Hisat2 (v2.0.5) (Kim et al., 2019). The mapped reads of each sample were assembled by StringTie (v1.3.3b) (Pertea et al., 2015). The featureCounts v1.5.0-p3 (Liao et al., 2014) was used to count the reads numbers mapped to each gene. Then the fragments per kilobase of exon per million mapped fragments (FPKM) of each gene was calculated based on the gene length and the reads count mapped to it. Differential expression analysis of two groups was performed using the DESeq2 R package (1.20.0) (Love et al., 2014). Padj < 0.05 and |log_2_(fold-change)| ≥ 1 were set as the threshold for significantly differential expression. ClusterProfiler (3.8.1) (Yu et al., 2012) was used for GO and KEGG enrichment analysis.The overall workflow for transcriptomic analysis is illustrated in **Supplementary Figure S1**.

### Validation of RNA-Seq data by qRT-PCR

Eight DEGs were randomly selected for qRT-PCR. The qRT-PCR reaction volume was 10 μl, containing 1 μl cDNA, 5 μl TB Green Premix Ex Taq II (Tli RNaseH Plus) (2×), 0.4 μl ROX Reference Dye II (50×), 0.8 μl of the forward and reverse primers, and 7 μl ddH_2_O. *Actin* was used as the endogenous reference gene. PCR reaction system: 95°C 30s; 95°C 5s, 60°C 34s, 40 Cycles; 95°C 15s, 60°C 1min, 95°C 15s. Primers were designed using Primer 5.0 (**Supplementary Table S1**). The application of the 2^–ΔΔC(t)^ method converts the instrument-generated threshold cycle value output into the relative gene expression level (Livak and Schmittgen, 2001). Three biological replicates were generated and three measurements were performed for each replicate.

## Results

### Different responses to submergence stress between Dianbei and Anba

**Figure 1:**
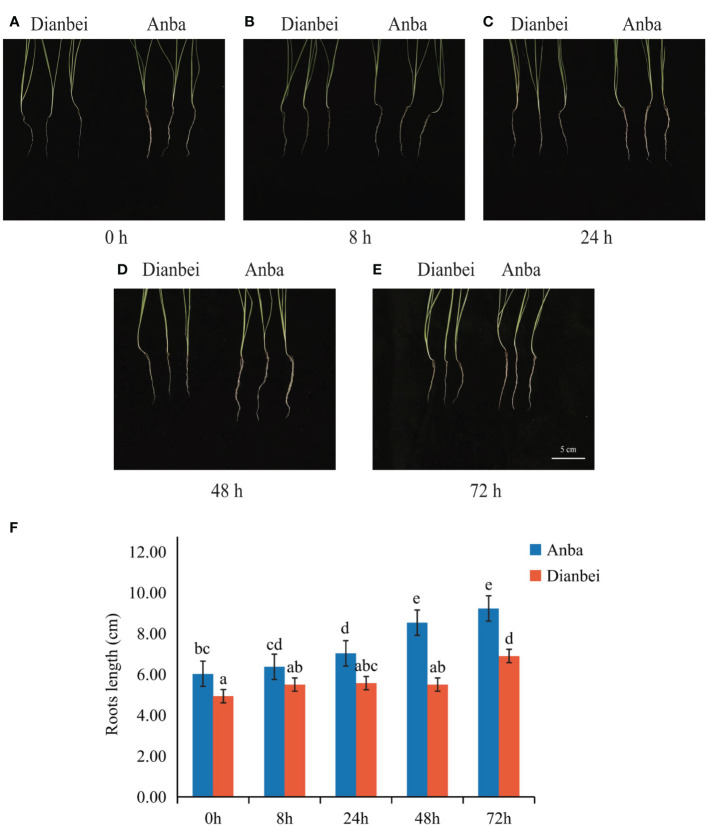
Root phenotype and length in Dianbei and Anba. **(A-E)** The phenotype of Dianbei and Aaba at 0 h **(A)**, 8 h **(B)**, 24 h **(C)**, 48 h **(D)**, and 72 h **(E)**. **(F)** Effects of submergence on the root lengths of Dianbei and Anba. Error bars indicate the standard error, n = 3. Different letters indicate the significant differences. Statistical analysis using one-way analysis of variance (ANOVA) using Duncan’s multiple range test (*P* < 0.05).

The morphologies of Dianbei and Anba under submergence stress were compared at 0 h, 8 h, 24 h, 48 h, and 72 h (**Figures 1A–E**). At 0 h, the root of Anba was 1.10 cm longer than Dianbei. After 72 h submergence stress, it was 2.33 cm longer than Dianbei. The roots of Dianbei and Anba gradually elongated with the increasing of submergence time. Anba showed a significant difference at 24 h, while Dianbei showed a significant difference at 72 h. As compared with 0 h, the roots of Dianbei and Anba increased post-submergence-stress by 39.96% and 53.07%, respectively (**Figure 1F**). Anba responded more positively to submergence stress. Thus, Dianbei is more resistant to submergence than Anba.

### Data analysis of RNA-Seq

**Figure 2:**
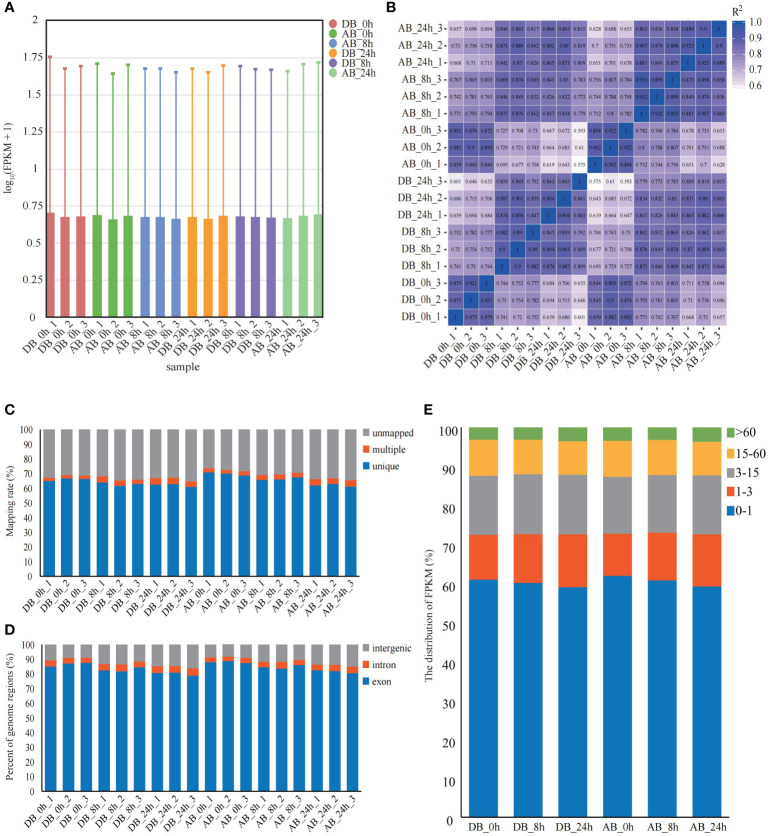
Mapping results of Dianbei and Anba. **(A)** The FPKM of Dianbei and Anba. The abscissa in the graph indicates different samples; the ordinate indicates the logarithmic values of the sample expression FPKM. **(B)** Pearson correlation between Dianbei and Anba. **(C-E)** Mapping ratio **(C)**, percent of genome regions **(D)**, and distribution of FPKM **(E)** in the roots of Dianbei and Anba under submergence stress.

To explore the gene expression and underlying molecular response mechanism of orchardgrass under short-term submergence stress. Illumina Novaseq 6000 was used to sequence the transcriptome of two orchardgrass cultivars roots at three different time points under submergence stress (0 h, 8 h, and 24 h), and 18 qualified RNA libraries were separately constructed (three library repeats for each time-point). After filtering out low-quality reads and reads containing N base, 792,201,680 clean reads and 118.82 Gb clean bases were obtained. For each sample, the Q30 values exceeded 93%, GC content was between 52.40 to 56.10%, and the error rate was only 0.03 (**Supplementary Table S2**). The distribution of gene expression levels for each sample was similar, and the overall gene expression level was high (**Figure 2A**, **Supplementary Figure S2**). The pearson correlation between samples was more than 0.8 (**Figure 2B**). Therefore, RNA-seq data was confirmed to be reliable and could be used in subsequent analyses.

HISAT2 software was used to map the clean reads to orchardgrass reference genome (Huang et al., 2020). Moreover, the clean reads average mapping rate was 68.18%, the unique mapping rate was between 60.92% to 70.88% (**Figure 2C**), and more than 78.8% of the clean reads were mapped to the exon region (**Figure 2D**). By comparing the FPKM of each sample, it was found that at each time point ~39.67% of the genes (DB_0h 39.05%, DB_8h 39.91%, DB_24h 40.96%, AB_0h 38.09%, AB_8h 39.21%, and AB_24h 40.8%) were expressed (FPKM ≥ 1), with over 3.10% of the genes were highly expressed (FPKM > 60) (**Figure 2E**).

### Differential expression analysis of Dianbei and Anba under submergence stress

**Figure 3:**
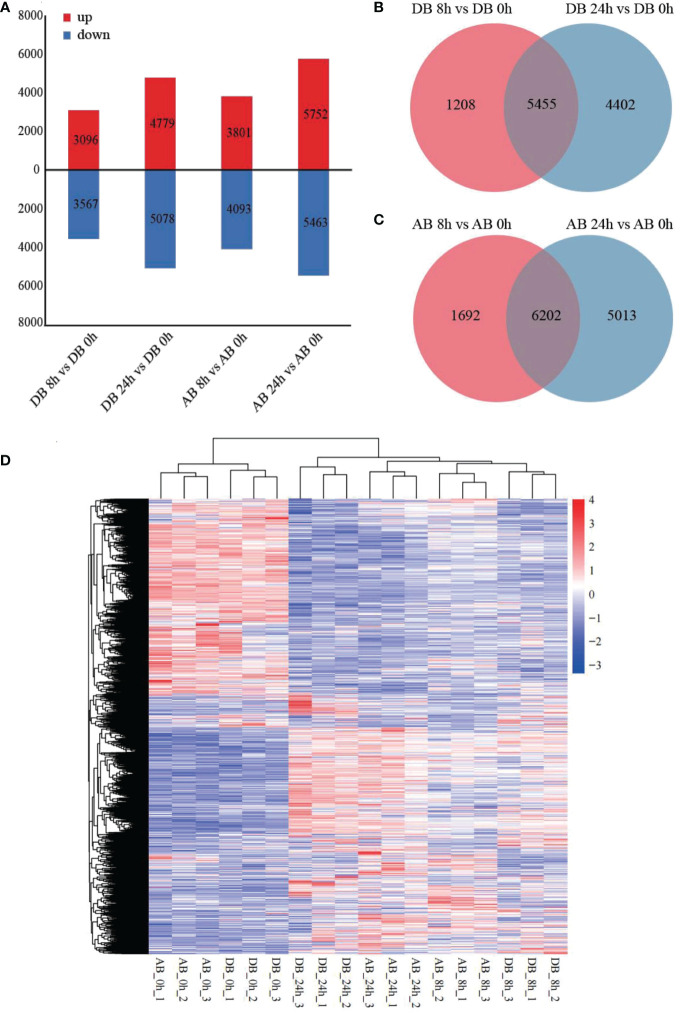
DEGs under submergence stress. **(A)** the number of up- and down-regulated genes in four pair-wise sampling stages, including DB 8 h vs DB 0 h, DB 24 h vs DB 0 h, AB 8 h vs AB 0 h, and AB 24 h vs AB 0 h **(B, C)** Venn analysis of the DEGs at 8 h and 24 h of post-submergence-stress induction as compared to control in Dianbei **(B)**, and Anba **(C)**. **(D)** the heatmap shows the respective expression levels of DEGs in each sample group, based on the average FPKM of biological replicates. The y-axis shows the cluster dendrogram of DEGs, and the x-axis shows the sample groups.

To explore the DEGs of Dianbei and Anba in response to submergence stress, the DEGs of two orchardgrass cultivars were screened with the thresholds of |log_2_ (fold-change)| > 1 and padj < 0.05. As compared with the control, a total of 6663 DEGs (3096 up-regulated and 3567 down-regulated) and 9857 DEGs (4779 up-regulated and 5078 down-regulated) were detected in Dianbei, while 7894 DEGs (3801 up-regulated and 4093 down-regulated) and 11215 DEGs (5752 up-regulated and 5463 down-regulated) were detected in Anba at 8 h and 24 h post-submergence-stress (**Figure 3A**, **Supplementary Figure S3**, **Supplementary Table S3**), respectively. Venn analysis was performed to examine the differential expression of roots tissue at different time points under submergence stress (**Figures 3B, C**). With 0 h as a control, 5455 and 6202 DEGs were found to be expressed in Dianbei and Anba at both 8 h and 24 h, respectively. With the increasing submergence stress time, the number of DEGs in the two orchardgrass cultivars gradually increased.

Gene cluster analysis of the two orchardgrass cultivars under submergence stress showed that Dianbei and Anba had similar expression patterns at the same stress time points, thus indicating that there were noticeable differences in the expression patterns of DEGs at different stress time points (**Figure 3D**). The differential expression of DEGs in Dianbei was higher than that in Anba at 8 h and 24 h post-submergence-stress, thus indicating that Dianbei, as a submergence-tolerant cultivar, has more DEGs for responding to submergence stress.

### GO and KEGG analysis of two orchardgrass cultivars DEGs under submergence stress

**Figure 4:**
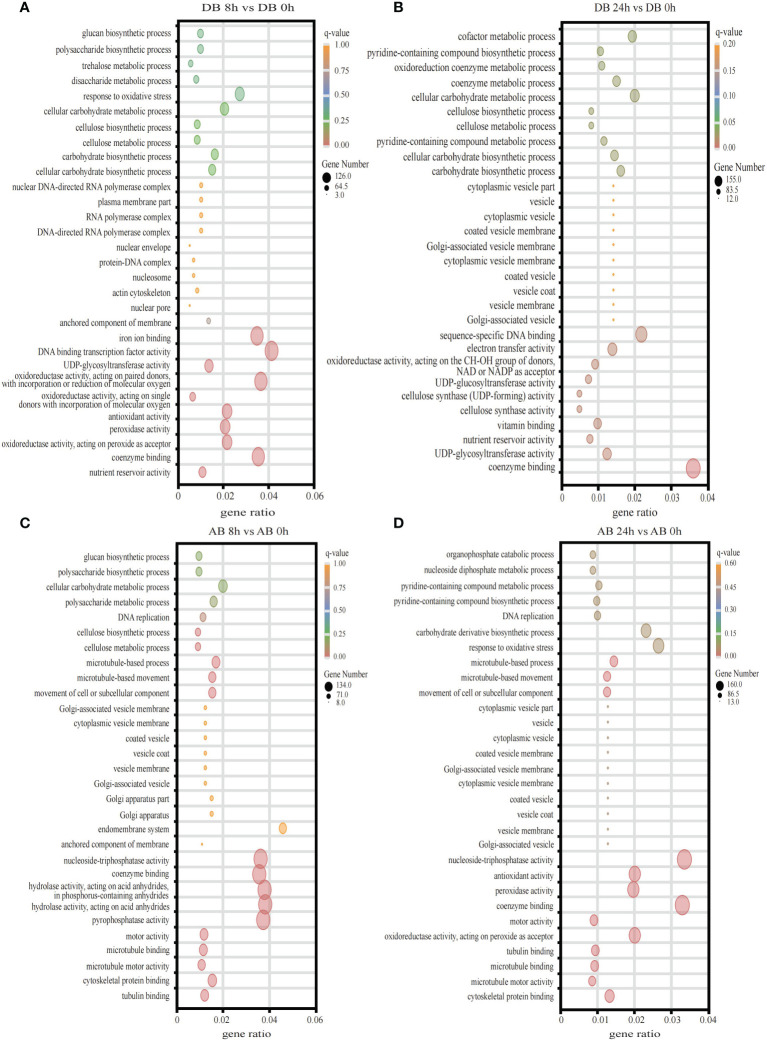
The GO enrichment analysis. **(A)** DB 8 h vs DB 0 h **(B)** DB 24 h vs DB 0 h **(C)** AB 8 h vs AB 0 h **(D)** AB 24 h vs AB 0 h The ordinate indicates the GO id, and the abscissa indicates the gene ratio. The size of the dot indicates the number of DEGs in the pathway, and the color indicates the different q-value.

To explore the functional significance of DEGs at different time points in Dianbei and Anba under submergence stress, we subjected GO enrichment analysis and obtained 986 terms. The DEGs were classified into three categories: biological processes (BP), molecular functions (MF), and cellular components (CC). The top 10 enriched terms of each category were selected for plotting. DEGs were mainly enriched in terms of biosynthesis and metabolism process, transcription factor activity, enzyme activity and coenzyme binding, oxidative response, microtubule, Golgi apparatus and vesicle (**Figure 4**, **Supplementary Table S4**). It is speculated that submergence stress may stimulate the expression of related genes in orchardgrass.

**Figure 5:**
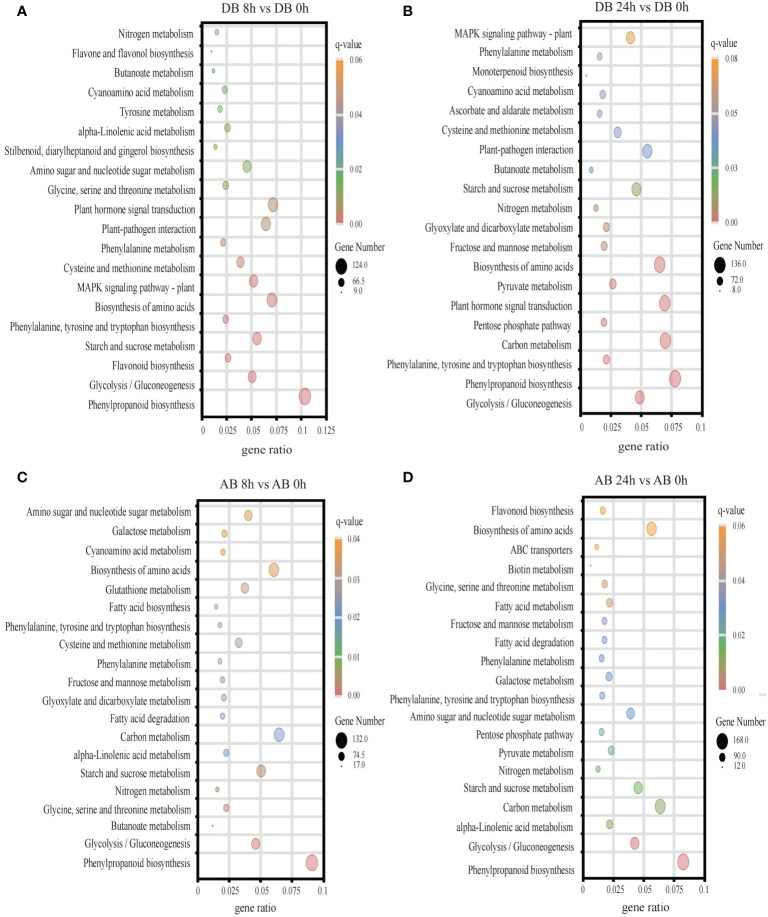
The KEGG pathway enrichment analysis. **(A)** DB 8 h vs DB 0 h **(B)** DB 24 h vs DB 0 h **(C)** AB 8 h vs AB 0 h **(D)** AB 24 h vs AB 0 h The ordinate indicates metabolic pathway, while the abscissa indicates the gene ratio. The size of the dot indicates the number of DEGs in the pathway, while the color indicates the different q-value.

In addition, KEGG enrichment analysis was performed and DEGs were mapped to 123 pathways (**Figure 5**, **Supplementary Table S5**). The results showed that pathways like phenylpropanoid biosynthesis, glycolysis/gluconeogenesis, and nitrogen metabolism were significantly enriched in both Dianbei and Anba. Enrichment of these pathways might indicate that submergence stress promoted their activation. It is worth mentioning that plant hormone signal transduction, MAPK signaling pathway, and plant-pathogen interaction were significantly enriched in Dianbei, but not in Anba. Therefore, it could be speculated that these pathways and genes might be related to the submergence tolerance in Dianbei and Anba.

### Plant hormone signal transduction in orchardgrass roots under submergence stress

**Figure 6:**
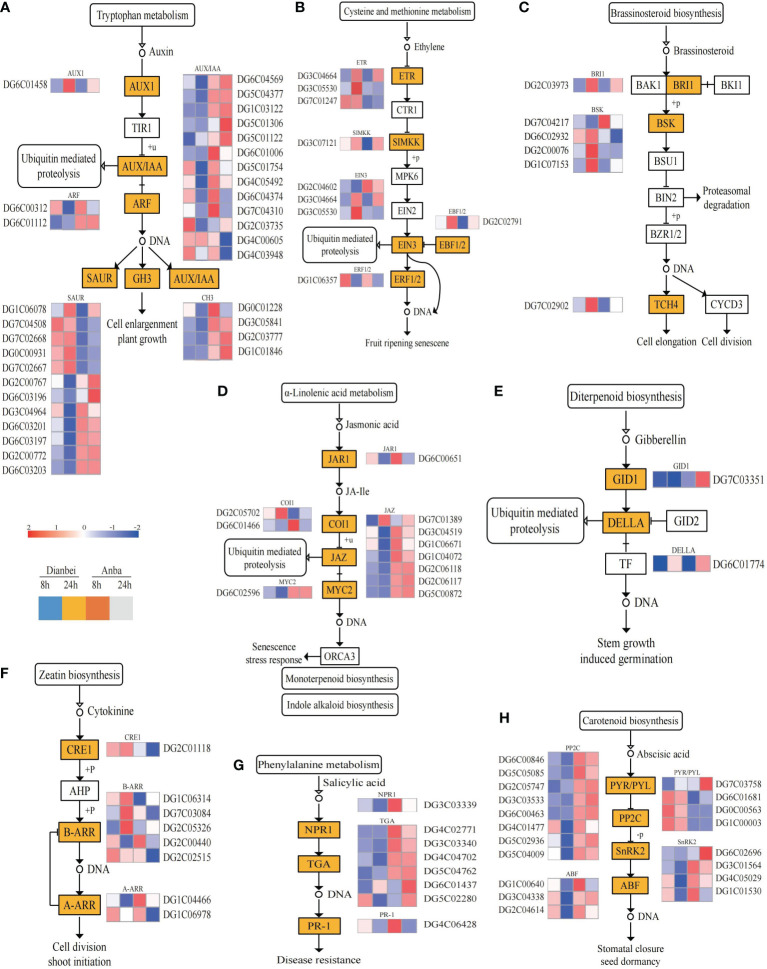
Plant hormone signal transduction pathway. **(A-H)** Auxin **(A)**, Ethylene **(B)**, Brassinosteroid **(C)**, Jasmonic acid **(D)**, Gibberellin **(E)**, Cytokinine **(F)**, Salicylic acid **(G)**, and Abscisic acid **(H)** biosynthesis pathway. The y-axis shows the cluster dendrogram of DEGs, and the x-axis shows the sample groups.

Plant hormones regulate plant growth and other biological processes as along with stress adaptation (Verma et al., 2016). In this study, the DEGs under submergence stress condition were analyzed for their KEGG pathway enrichment (**Figure 5**). The results showed that submergence stress activated various plant hormones (auxin, cytokinin, ethylene, abscisic acid (ABA), jasmonic acid (JA), salicylic acid (SA), brassinosteroids (BR), gibberellin (GA), etc.) signal pathways in orchardgrass roots (**Figure 6**, **Supplementary Table S6**). In Dianbei, multiple genes related to ARF and GH3 in auxin, CRE1 in cytokinin, GID1 in GA, PP2C, SnRK2 and ABF in ABA, and NPR1 in SA, were down-regulated at 8 h and 24 h post-submergence-stress, triggering speculation that these genes were inhibited under submergence stress. Multiple genes related to PYR/PYL in ABA, ETR and EBF1/2 in ethylene, and BRI1 in BR, were up-regulated at 8 h and 24 h post-submergence-stress, indicating that these genes were activated under submergence stress. Additionally, multiple genes related to AUX1 in auxin, DELLA in GA, SIMKK in ethylene, and TCH4 in BR, were up-regulated at 24 h, and PR-1 in SA, JAR1 and MYC2 in JA, were down-regulated at 24 h, but had no significant change at 8 h post-submergence-stress. These results indicate that these genes may need a certain time to respond to submergence stress. Most DEGs were down-regulated post submergence stress. Thus, we speculated that submergence stress may inhibit plant hormones signal transduction, the inhibitory effect increased with the increasing submergence time. In addition, among the DEGs involved in plant hormone signal transduction, only 10 DEGs (DG2C00767, DG2C00772, DG2C01118, DG2C02515, DG2C06118, DG4C03948, DG5C01306, DG5C01122, DG6C01437, and DG7C03758) had higher differential expression in Anba than in Dianbei, while the other DEGs showed the opposite, thus indicating that the differential expression of these genes may be one of the reasons for the different submergence tolerance capabilities between Dianbei and Anba.

### MAPK signal transduction in orchardgrass roots under submergence stress

**Figure 7:**
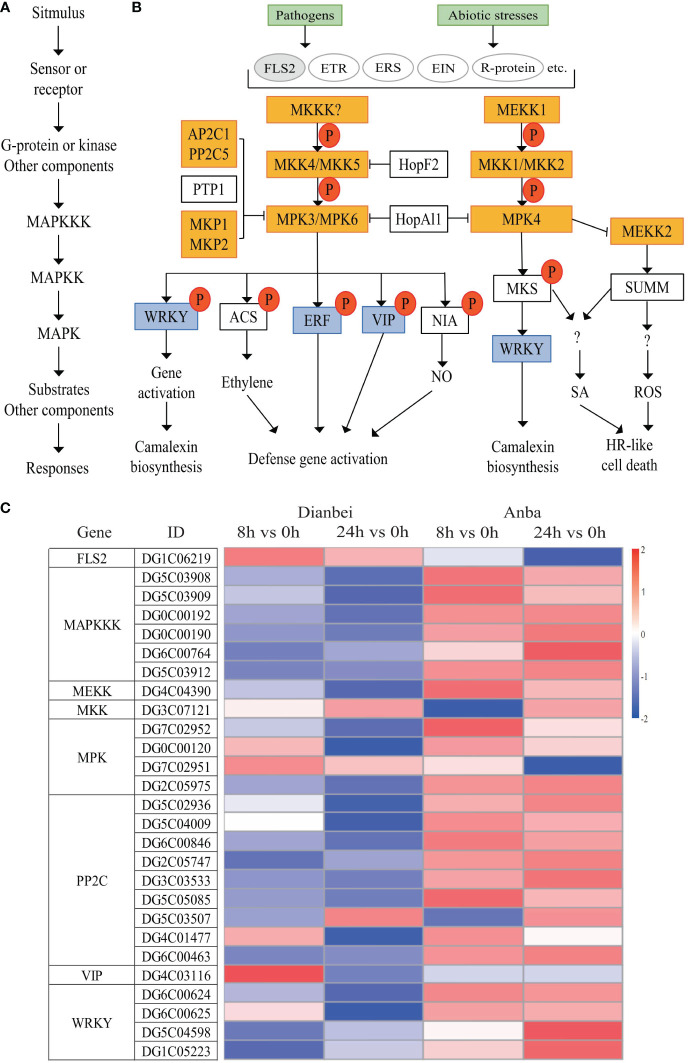
MAPK signal transduction pathway. **(A)** Plant MAPK cascades pattern. **(B)** Possible functions of various MAPK cascades in plants. **(C)** Heatmap of DEGs involved in the MAPK signaling pathway. The y-axis shows the cluster dendrogram of DEGs, and the x-axis shows the sample groups.

MAPK signal transduction is one of the most well-studied plant signaling mechanisms, and plant MAPK cascades play pivotal roles in signaling plant defense against biotic and abiotic stresses. (Zhang and Klessig, 2001). The MAPK cascade is minimally composed of different combinations of at least three protein kinases: MAPKKK (MAP3K/MEKK/MKKK), MAPKK (MKK/MEK), and MAPK (MPK), which activate each other sequentially *via* phosphorylation (Danquah et al., 2014) (**Figures 7A, B**). In this study, DEGs related to MAPK signal transduction, FLS2 was up-regulated and activated the transmembrane transport of flg22, while most DEGs related to MAPK cascade signal were down-regulated, thus indicating that submergence stress may inhibit the amplification of the MAPK cascade signal. Two DEGs, DG1C06357 and DG1C04245, showed significant differential expression at 8 h, but had no significant change at 24 h of post-submergence-stress, thus indicating that these two DEGs may play an important role in the early submergence stress response. The differential expression of other DEGs showed an increasing trend with the increasing submergence time. Out of these DEGs, seven DEGs (DG6C02930, DG2C04602, DG3C07121, DG6C02696, DG4C06428, DG5C04434, and DG4C03116) had no significant change at 8 h, but showed significant differential expression at 24 h post-submergence-stress, thus indicating that these genes may need some time to respond to submergence stress. In addition, eight DEGs (DG0C00183, DG0C00192, DG0C00190, DG5C03912, DG2C05975, DG6C00846, DG6C00463, and DG1C05223) were differentially expressed at 8 h and 24 h post-submergence-stress in Dianbei, while showing no significant expression in Anba, thus indicating that these DEGs may be one of the reasons for the different submergence tolerance abilities between Dianbei and Anba (**Figures 7B, C**, **Supplementary Table S6**).

Additionally, studies have been shown that MAP kinases are involved in ABA-mediated stomatal closure. The active SnRK2 kinase phosphorylates the NADPH oxidase RbohF, leading to ROS accumulation. The ROS-mediated activation of MAPKs positively regulates the ABA-mediated stomatal closure (Danquah et al., 2014). In this study, three types of DEGs were found to participate in this pathway (**Figures 6H**, **7C**). Among them, the genes related to PYR/PYL receptor proteins were up-regulated, while the genes related to PP2C and SnRK2 kinase biosynthesis were down-regulated. Dianbei showed a greater variation than Anba, and the differential expression of DEGs increased with the increasing submergence time. It indicated that submergence stress activated the PYR/PYL expression in the upstream, and inhibited the expression of PP2C and SnRK2 in the downstream of ABA pathway, thus inhibiting ABA-mediated stomatal closure and increasing the respiration of orchardgrass under submergence stress, and promoting more oxygen uptake. Therefore, Dianbei can respond more quickly and positively to submergence stress, which may be one of the reasons for the different submergence tolerance between Dianbei and Anba.

### Ca^2+^ signal transduction in orchardgrass roots under submergence stress

**Figure 8:**
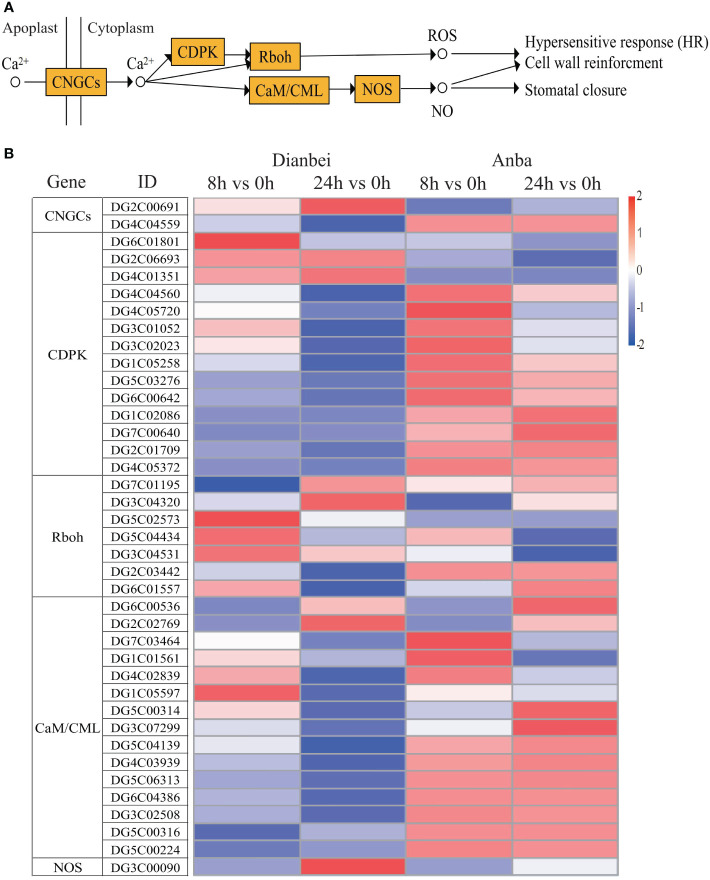
Ca^2+^ signal transduction pathway. **(A)** Schematic diagram of Ca^2+^ signaling pathway. **(B)** Heatmap of DEGs involved in Ca^2+^ signaling pathway. The y-axis shows the cluster dendrogram of DEGs, while the x-axis shows the sample groups.

During the KEGG pathway enrichment analysis under submergence stress in orchardgrass, it was found that in plant-pathogen interaction signal transduction, 39 DEGs also participated in the Ca^2+^-mediated hypersensitive response (HR) besides MAPK signal transduction pathway (**Figure 8**, **Supplementary Table S6**). Similar to the plant hormone and MAPK signal transduction pathways, the differential expression of DEGs increased with the increase of submergence time except for DG4C04884 and DG2C02769. The amplitude of variation in Dianbei was greater than that in Anba, which again indicated that Dianbei could respond more rapidly and positively than Anba under submergence stress. Interestingly, the genes related to Ca^2+^ transmembrane CNGCs receptor proteins and NOS (NO synthesis rate limiting enzymes) were up-regulated, which activated Ca^2+^ transmembrane transport, NO synthesis, and induced HR production in orchardgrass under submergence stress.

### Validation of transcriptome sequencing data by qRT-PCR

**Figure 9:**
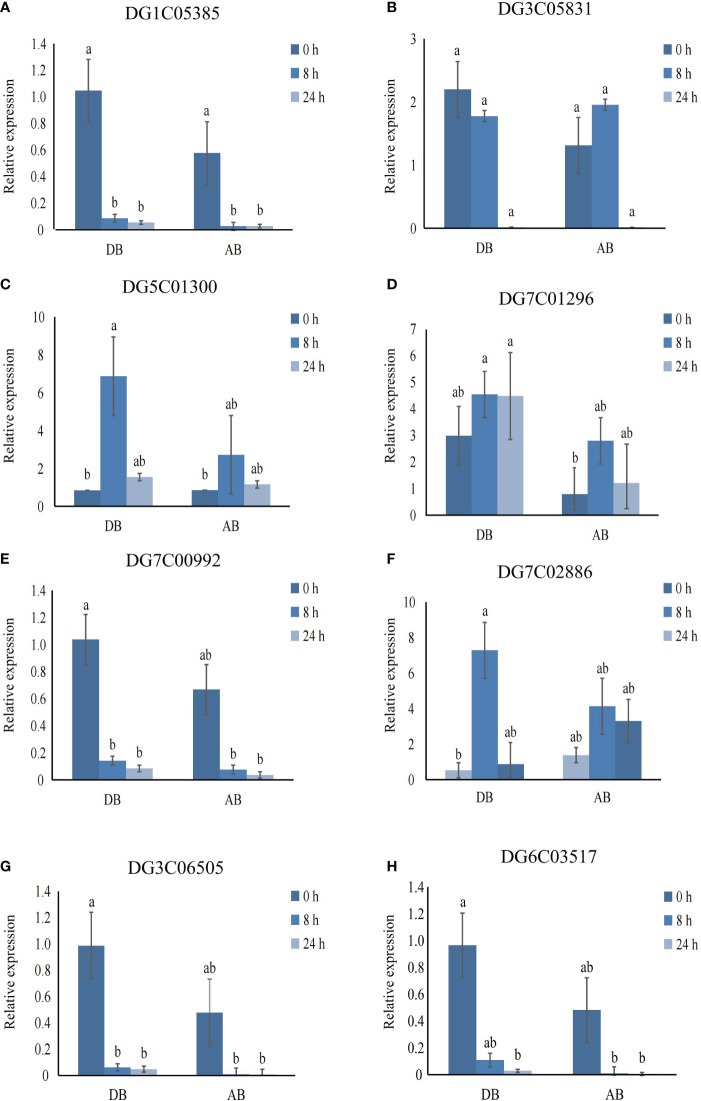
Validation of genes expression using qRT-PCR. **(A)** DG1C05385. **(B)** DG3C05831. **(C)** DG5C01300. **(D)** DG7C01296. **(E)** DG7C00992. **(F)** DG7C02886. **(G)** DG3C06505. **(H)** DG6C03517. Error bars indicate the standard error, n = 3. Different letters indicate the significant differences. Statistical analysis using one-way analysis of variance (ANOVA) using Duncan’s multiple range test (*P* < 0.05).

To verify the reliability of transcriptome results, we selected eight genes for qRT-PCR (**Figure 9**). The expression profiles of these genes were consistent with the sequencing results, indicating that our analysis based on transcriptome data is reliable.

## Discussion

**Figure 10:**
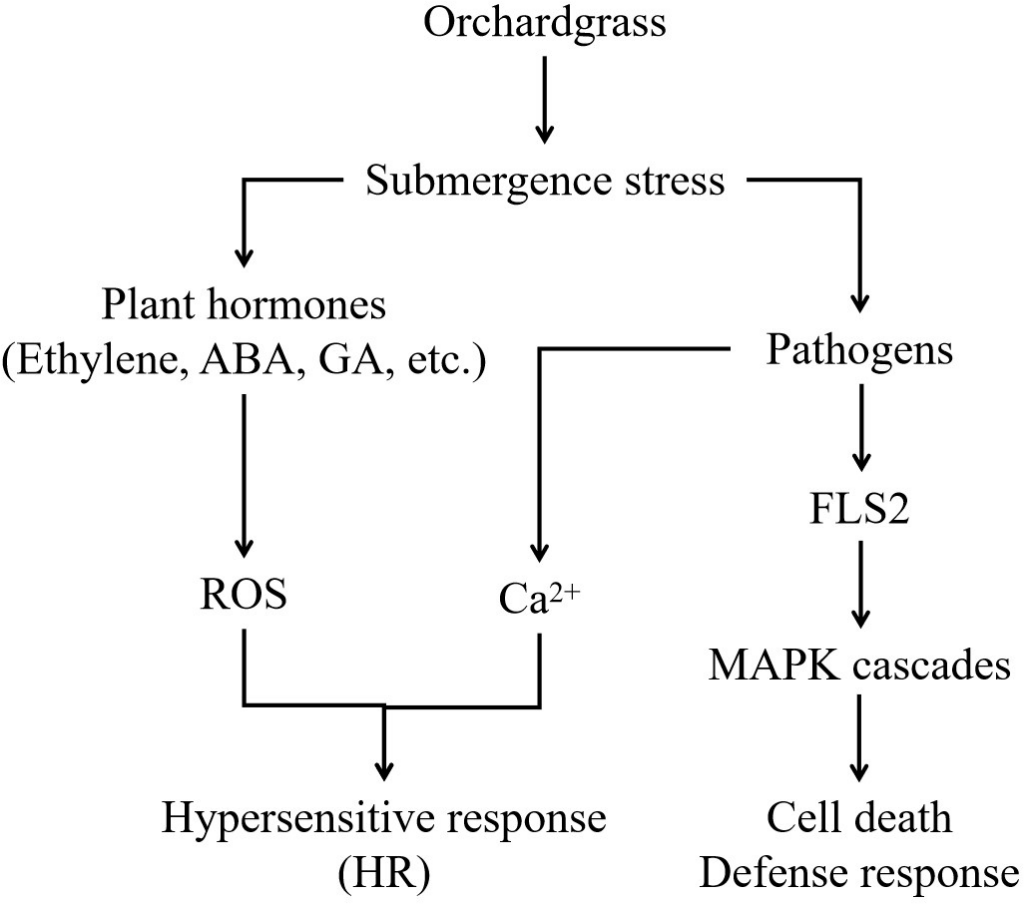
A proposed model for orchardgrass under submergence stress. The figure depicts the interplay and crosstalk of plant hormones, pathogen interaction, and MAPK cascades in orchardgrass during submergence stress.

Water is essential for all plants, but excessive water or submergence results in stress and prevents gaseous exchange between the soil and atmosphere. Excessive water can inhibit the growth and development and even lead to death. In this study, a model was constructed by transcriptome analysis to understand the response of orchardgrass under submergence stress (**Figure 10**).

In one day of waterlogging, the partial pressure of O_2_ fell from 20.9 to 1 kPa, leading to anoxia (Phukan et al., 2016). To self-repair and maintain stability, tissues or organs specific functions cells require the mitochondrial oxidative phosphorylation of ATP (Phukan et al., 2016). Therefore, in order to survive the energy crisis in waterlogged or submerged conditions, ATP related processes, like DNA synthesis and cell division, are inhibited (Gibbs and Greenway, 2003). In this study, we found that DEGs of two orchardgrass cultivars were significantly expressed in biosynthesis and metabolism process, transcription factor activity, enzyme activity and coenzyme binding, oxidative response and cellular components (microtubules, Golgi, and vesicles) terms under submergence stress (**Figure 4**). Therefore, the results indicated that the synthesis and metabolism of intracellular molecules are important in the submergence stress response of orchardgrass.

Waterlogging reduces the soil permeability, inhibits the plant TCA cycle, promotes the fermentation of the glycolysis-generated pyruvate acid to produce ethanol and NAD^+^, thus altering the energy metabolism and causing the accumulation of toxic substances in roots (Judge and Dodd, 2020). Glycolysis and galactose metabolism are important glucose metabolism processes in plants, which provide energy for the plant growth and development. Strengthening the glycolysis pathway can alleviate the hypoxia-induced energy shortage under waterlogging stress (Kolahi et al., 2021). In quinoa (*Chenopodium quinoa* Willd.), the expression of saccharides and alcohol-related genes in Dianli-1299 (waterlogging-resistant cultivar) increased (Guo et al., 2022). Nitrogen application can effectively improve the development of rapeseed roots and reduce the waterlogging stress-induced damage (Men et al., 2020). In this study, it was found that, phenylpropanoid biosynthesis, glycolysis/gluconeogenesis, and nitrogen metabolism were significantly enriched in two orchardgrass cultivars under submergence stress (**Figure 5**), indicating that phenylpropanoid biosynthesis, glycolysis/gluconeogenesis and nitrogen metabolism participate in maintaining the balance of energy metabolism in orchardgrass roots under submergence stress. In addition, plant hormone signal transduction, MAPK signaling pathway, and plant-pathogen interaction were significantly enriched in Dianbei, but not in Anba (**Figure 5**). It is speculated that these pathways might be related to the submergence tolerance in Dianbei and Anba.

Plant hormones mediate plants abiotic stress response (Shu et al., 2018). The interaction of plant hormones is crucial in waterlogging and submergence tolerance. Some plant endogenous hormones, like abscisic acid, ethylene, auxin and cytokinin, are very sensitive to waterlogging stress and can effectively alleviate its adverse effects (Bashar, 2018; Qi et al., 2019; Hu et al., 2020). The level of ethylene increases in waterlogged plants, which has been identified as a signal that regulates the early response to submergence stress (Phukan et al., 2016). ERF transcription factors are regulated by ethylene. Exogenous ethylene significantly promotes ERF transcription activity in *Arabidopsis* and soybean (*Glycine max* L.) (Hess et al., 2011; Tamang et al., 2014). ETR1 activates EIN2/EIN3, which induces the expression of ERF transcription factors, and promotes ethylene production under waterlogging stress (Pierik et al., 2006). Plants, such as rice, which slowly produces ethylene in its roots, positively respond to ethylene. Ethylene reduces the level of ABA level and enhances the sensitivity of GA in tissue (van der Knaap and Kende, 1995). In rice, *Sub1A* inhibited the accumulation of DELLA, a GA-responsive protein, and prolonged the submergence tolerance time by preserving ATP and CHO content (Peña-Castro et al., 2011). The endogenous ABA concentration did not decrease under flooding in soybean, but it was decreased in untreated plants. The transcriptional expression system in soybean which provided exogenous ABA was better, thus indicating that exogenous ABA enhanced the waterlogging tolerance in soybean (Komatsu et al., 2013). In this study, in Dianbei, the genes related to ETR, SIMKK, EIN3, and EBF1/2 in Ethylene were up-regulated, GID1 and DELLA in GA were down-regulated, and PYR/PYL were up-regulated, which inhibited the downstream expression of PP2C, SnRK2, and ABF in ABA under submergence stress (**Figure 6**). This indicates that submergence stress stimulates ethylene biosynthesis and metabolism, and reduces ABA and GA in Dianbei. These results were similar to the results in rice and soybean under waterlogging stress (Peña-Castro et al., 2011; Komatsu et al., 2013). Most DEGs related to plant hormone signal transduction were differentially expressed in Dianbei, and the differential expression was down-regulated with the increasing submergence treatment time (**Figure 6**). Therefore, we speculate that the differential expression of these genes might be one of the reasons for the different submergence tolerance in Dianbei and Anba. In addition, the submergence stress may inhibit plant hormone signal transduction, which increases with the increasing submergence time.

Under waterlogging stress, an efficient carbohydrate mobilization mechanism in plant roots enables cells to survive prolonged hypoxia (Phukan et al., 2016). Cytosolic Ca^2+^ increased rapidly in maize and *Arabidopsis*, which ultimately altered the expression of hypoxia-responsive genes in hypoxia conditions (Subbaiah and Sachs, 2003; Bailey-Serres and Chang, 2005). Hypoxia leads to ROS production, in turn, ROS signal transduction promotes various defense responses (Steffens et al., 2012). In *Arabidopsis*, ROS activates MAPK6 to improve the survival under hypoxic conditions. (Chang et al., 2012). However, excessive ROS causes oxidative damage. Therefore, the ROS defense and signaling cascade are strictly regulated in plants to maintain an appropriate balance between survival and stress tolerance (Sachdev et al., 2021). Waterlogging could induce MAPK cascades, promote the regulation of plant hormones, and forme aerenchyma tissues, which modulates the morphological adaptations in maize roots (Kaur et al., 2021). In *Arabidopsis*, MAPK led to phosphorylation of ACC synthase and participated in aerenchyma formation (Liu and Zhang, 2004). In this study, CNGCs, Ca^2+^ transmembrane transduction-mediating receptor proteins, were up-regulated and activated Ca^2+^ transmembrane transport in orchardgrass under submergence stress. Most genes in MAPK signal transduction were down-regulated, which inhibited the amplification of MAPK cascade signaling (**Figures 7**, **8**). Moreover, with the increasing submergence time, the amplitude of differential expression of genes gradually increased, and Dianbei was greater than Anba. Therefore, we speculate that the differential expression of these genes might be one of the reasons for the different submergence tolerance abilities in Dianbei and Anba.

## Conclusion

In this study, we tested the morphology indexes of roots of two orchardgrass cultivars at 0 h, 8 h, 24 h, 48 h and 72 h. Simultaneously, we obtained 118.82 Gb clean data from RNA-seq analysis of orchardgrass roots. At 8 h and 24 h post-submergence-stress, Dianbei identified 6663 and 9857 DEGs, while Anba identified 7894 and 11215 DEGs, respectively. With 0 h as a control, 5455 and 6202 DEGs were differentially expressed at 8 h and 24 h in Dianbei and Anba, respectively. These results indicate that Anba has more genes to respond to submergence stress. Moreover, genes related to biosynthesis and metabolism, cellular component, transcription factor activity, enzyme activity and coenzyme binding, phenylpropanoid biosynthesis, glycolysis/gluconeogenesis, and nitrogen metabolism were involved in the submergence stress response in orchardgrass. The expression of genes involved in plant hormone, MAPK, and Ca^2+^ signal transduction were significantly in Dianbei, but not in Anba. The differential expression of these genes and pathways may be the main reasons behind the different submergence tolerance abilities in Dianbei and Anba. Since the submergence tolerance of orchardgrass is controlled by multiple genes, RNA-seq can be used for comprehensively exploring submergence tolerance-related genes and pathways. Therefore, the study can facilitate further understanding of the molecular regulatory mechanism in orchardgrass roots under submergence stress conditions.

## Data availability statement

The datasets presented in this study can be found in online repositories. All datasets can be found in the article/**Supplementary Material**.

## Author contributions

BZ (teacher, corresponding author), YF, MQ, and YZ conceived and designed the project and the strategy. MQ, LB, and BZ (student, co-author) contributed to plant samples collection. MQ, XY, and PS performed experiments. MQ, BS, and WL analyzed data. BZ (teacher, corresponding author), YF, MQ, and XZ wrote the manuscript.

## Supplementary material

Supplementary Table 1 The Primer design of genes.

Supplementary Table 2 Transcriptome analysis of 18 orchardgrass roots.

Supplementary Table 3 The list of differentially expressed genes.

Supplementary Table 4 The list of GO enrichment.

Supplementary Table 5 The list of KEGG pathway enrichment.

Supplementary Table 6 The log_2_(fold-change) in plant hormone, MAPK, and Ca^2+^ signal transduction pathway.
