# Supplementary material for: Comparative transcriptomic analysis of the gene expression and underlying molecular mechanism of submergence stress response in orchardgrass roots
Source: Front Plant Sci. 2023 Jan 10;13:1104755. doi: 10.3389/fpls.2022.1104755 (PMC9871833; doi:10.3389/fpls.2022.1104755)
Supplement: Supplementary file 7 [file DataSheet_1.docx]

Supplementary Material


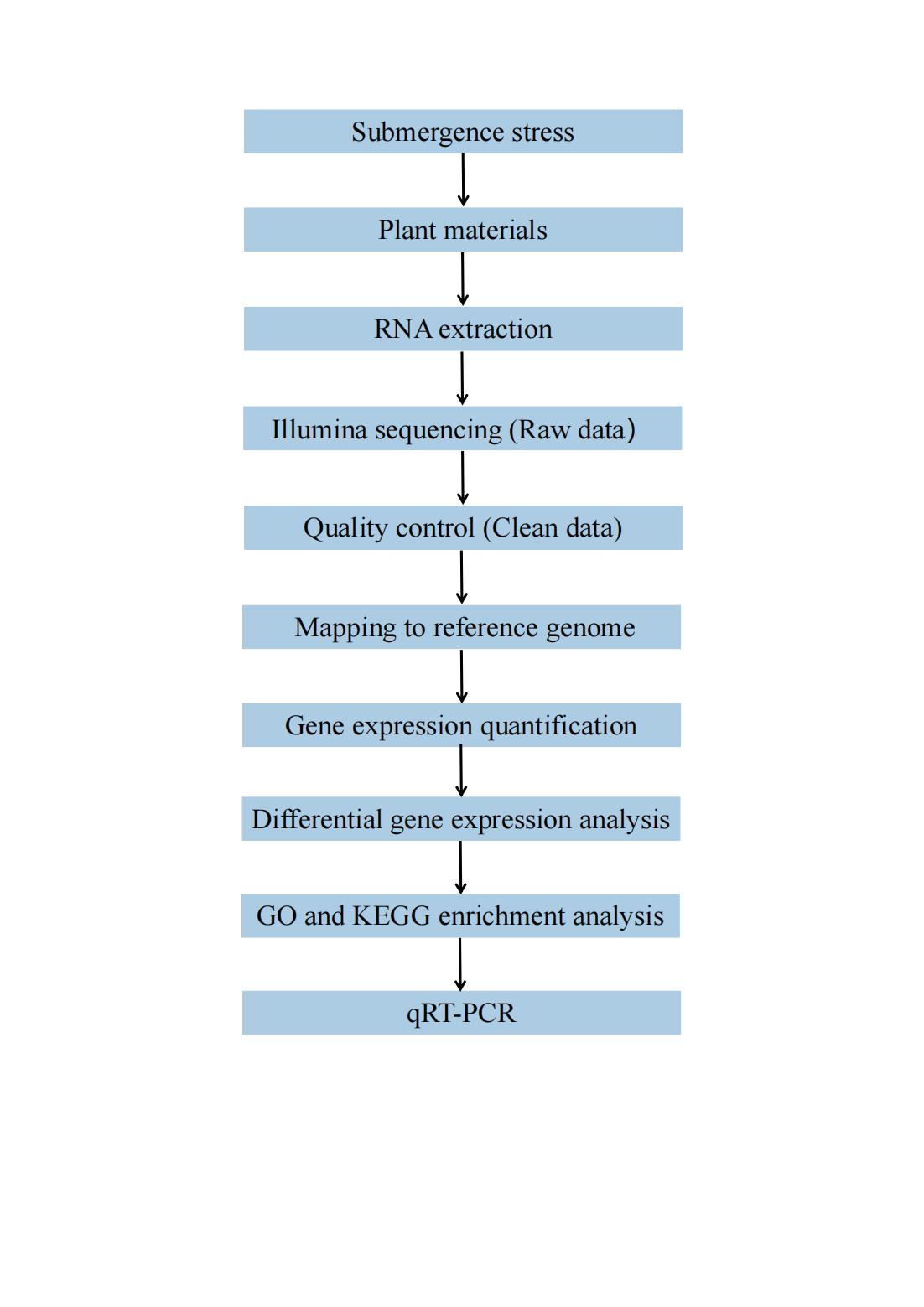


**Supplementary Figure S1. The workflow for transcriptomic analysis.**


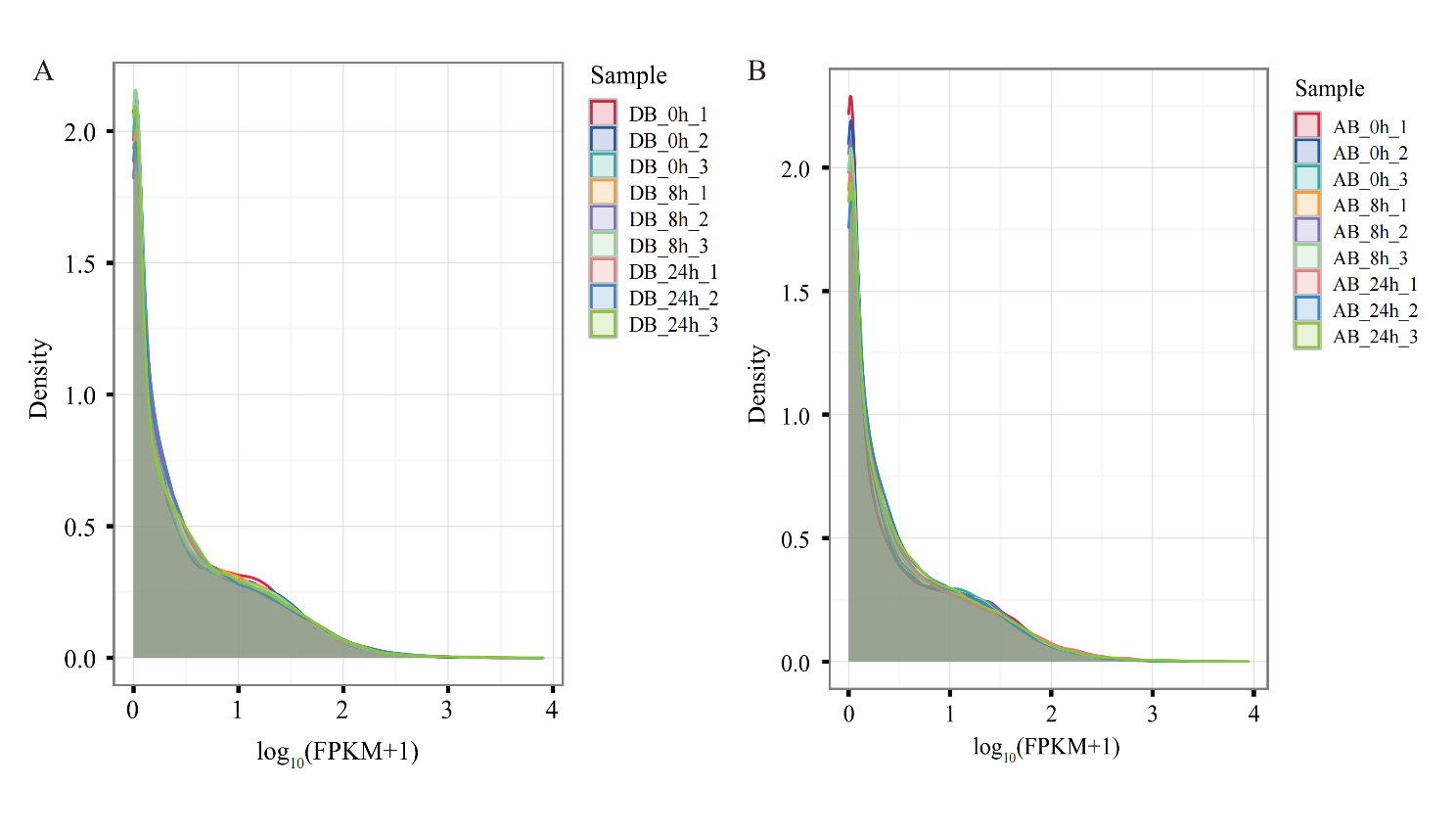


**Supplementary Figure S2. Expression density distribution plot.** The density plots of Dianbei **(A)** and Anba **(B)**. The curves of different colors in the graph represent different samples; the abscissas of the points on the curve indicate the logarithmic values of FPKM of the corresponding samples, and the ordinate of the points indicates the probability density.


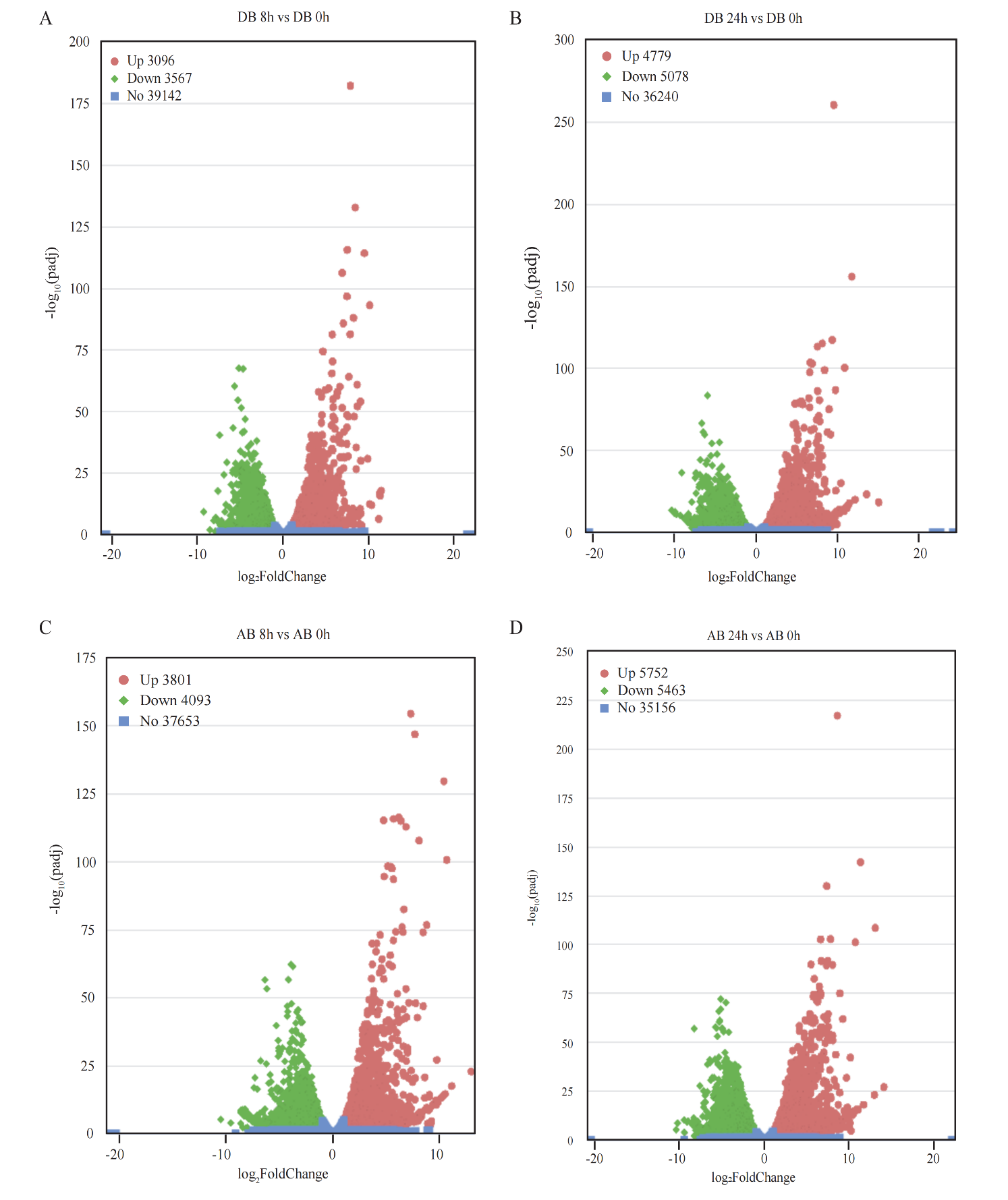


**Supplementary Figure S3. Differential gene distribution volcano plot. (A)** DB 8 h vs DB 0 h. **(B)** DB 24 h vs DB 0 h. **(C)** AB 8 h vs AB 0 h. **(D)** AB 24 h vs AB 0 h.
